# Editorial: Sentinels of health: advancements in monitoring and surveillance of vector-borne diseases in domestic and wild animals and vectors

**DOI:** 10.3389/fvets.2025.1670316

**Published:** 2025-08-13

**Authors:** Vesna Milićević, Francesco Mira, Mihaela Kavran

**Affiliations:** ^1^Virology Department, Institute of Veterinary Medicine of Serbia, Belgrade, Serbia; ^2^Istituto Zooprofilattico Sperimentale della Sicilia “A. Mirri”, Palermo, Italy; ^3^Faculty of Agriculture, Centre of Excellence—One Health, University of Novi Sad, Novi Sad, Serbia

**Keywords:** vector-borne diseases, surveillance, zoonotic pathogens, One Health, vector ecology, integrated control strategies

Vectors and vector-borne diseases in domestic and wild animals are undergoing significant shifts in distribution and impact due to environmental and climate changes, globalization, and human encroachment on natural habitats. Formerly localized, many of these pathogens are now spreading across new regions, with several acquiring zoonotic potential and posing public health risks. These diseases also impose substantial economic burdens through veterinary costs, reduced productivity, and control efforts.

Recognizing the complexity of these challenges, *Frontiers in Veterinary Science* launched the Research Topic “*Sentinels of health: advancements in monitoring and surveillance of vector-borne diseases in domestic and wild animals and vectors*” in February 2024. This initiative aimed to consolidate current knowledge on surveillance, vector ecology, pathogen detection, and control strategies, emphasizing a multidisciplinary and One Health approach.

The contributions highlight advances in molecular diagnostics, phylogenetics, ecological modeling, and field surveillance, demonstrating how science is improving our capacity to the early detection and to monitor and respond to emerging threats.

Strengthening epidemiological systems and understanding the ecological drivers of vector dynamics remain essential for early outbreak prediction and effective intervention. This Research Topic underscores the urgent need for coordinated, adaptive strategies to manage the evolving landscape of vector-borne and zoonotic diseases.

A multi-year surveillance study conducted by Ivović et al. in Slovenia provided critical insights into the ecology of sandflies, including *Phlebotomus papatasi, P. neglectus, P. perniciosus*, and *P. mascittii*. The survey, covering 43 sampling sites from 2020 to 2022, recorded peak sandfly activity in July and showed a strong correlation between sandfly distribution and climatic variables such as temperature and precipitation. Although *Leishmania* spp. and *Phleboviruses* were not detected, predictive habitat models underlined the significance of ongoing monitoring in the face of climate change. Continuous surveillance remains vital for early detection of shifts in vector populations and for assessing future pathogen emergence.

Complementing ecological investigations, Vaselek and Alten explored the gut microbiome of *Ph. papatasi*, emphasizing the crucial role of microbial communities in larval development. Their results showed that larvae reared in microbiome-depleted conditions had higher mortality rates and delayed development compared to those raised on media with live, diverse microbiota. These findings suggest that manipulating sandfly microbiota could potentially be employed in biological control strategies. Additionally, the influence of the gut microbiome on *Leishmania* development within the sandfly vector has been previously demonstrated (1), underscoring the microbiome's role in vector competence.

Surveillance of zoonotic pathogens in domestic ruminants was addressed by Barbic et al., who conducted a seroprevalence study in sheep from endemic areas of Croatia. Historically, chickens and horses were used as sentinels for detecting seasonal WNV incursions, correlating with human outbreaks (2). This study expanded sentinel species to include sheep, revealing exposure to TBEV (9.7%), WNV (3.0%), *Borrelia burgdorferi* s.l. (2.7%), and USUV (1.3%). Significant associations were observed with micro-location, sheep breed, and farm distance from households. The data support the inclusion of sheep in integrated surveillance networks and call for targeted prevention strategies based on local epidemiological factors.

In a comprehensive study from Vietnam, Do et al. reported a 29% prevalence of *Rhipicephalus sanguineus* infestation in client-owned dogs. Pathogen screening of tick pools revealed high infection rates with *Mycoplasma* spp. (78.5%), *Anaplasma* spp. (37.3%), and *Rickettsia felis* (5.1%). Seasonal patterns and host factors such as age, breed, and lifestyle were associated with tick infestation risk. These results underscore the zoonotic potential of tick-borne pathogens in companion animals and the need for integrated tick control, owner education, and surveillance.

Addressing inaccuracies in host-vector records, Millot et al. critically reevaluated the literature on *Culicoides* biting midges in France. While previous data suggested that 92 species fed on cattle, their molecular and indirect investigations confirmed feeding behavior in only 45 species. This correction highlights the importance of combining morphological and molecular tools in determining vector-host interactions, which is essential for accurate risk assessments of vector-borne diseases.

In the field of veterinary diagnostics, Li et al. developed and validated a multiplex quantitative PCR assay capable of simultaneously detecting six major pathogens associated with the bovine respiratory disease complex: BRSV, BPIV3, BVDV, BAV3, *Mycoplasma bovis*, and IBRV. The assay demonstrated high specificity, sensitivity, and reproducibility, with detection limits ranging from 4.99 to 74.4 copies/μl. Among 224 clinical samples, a 25% mixed infection rate was observed, illustrating the assay's utility in complex diagnostic scenarios.

Refinements in environmental sampling for ASFV surveillance were investigated by Kwon et al., who compared multiple swabbing devices and preservation techniques on clean stainless steel surfaces. Their results showed that pre-moistened gauze, sponge sticks, and nucleic acid preservatives significantly improved ASFV DNA detection compared to dry swabs. These optimized methods can enhance early outbreak detection and inform disinfection protocols on farms.

While Glišić et al., (3) showed that the human stands for the main risk of disease spreading, Vasić et al. examined alternative ASFV transmission routes during the 2023 outbreak in Serbia. While no viral DNA was detected in soil, feed, or barn swabs, positive results were obtained from fly larvae and adults (*Lucilia sericata, Stomoxys calcitrans*) collected from carcasses. Experimental infections confirmed short-term ASFV persistence in *S. calcitrans*. These findings suggest a potential mechanical transmission pathway and highlight the role of insect vectors in outbreak dynamics.

Šolaja et al. focused on the genetic evolution of WNV in the Western Balkans through whole-genome sequencing of field strains. Phylogenetic analysis revealed two major sublineages within the E clade (E1 and E2), distinct from previously defined D and E clades (4). Variants in E159, NS1, and NS3 proteins were identified, with potential roles in virulence and immune evasion. These findings demonstrate the continued evolution of WNV and underscore the need for molecular surveillance to track viral adaptation.

In a separate case of emerging viral threat, Hu et al. documented an outbreak of recombinant LSDV among yaks on the Qinghai-Tibet Plateau. The strain, classified within Cluster 1.2 recombinant subclade, showed high similarity to Southeast Asian LSDV isolates and induced systemic lesions and 46.7% mortality. Histopathology and immunohistochemistry confirmed viral replication in epithelial and immune cells. The study illustrates how recombinant viruses can overcome ecological barriers and emphasizes the necessity of high-altitude-specific vaccination and surveillance strategies.

In geographically isolated ecosystems, Zanella et al. screened 411 equids in the Galapagos Islands for WNV, USUV, and EIAV. All samples tested negative, indicating the current absence of these pathogens. However, the presence of competent vectors and increasing human and animal movement necessitates continued surveillance to prevent pathogen introduction in these ecologically sensitive environments.

Warm climates in Africa, southern Europe, and Southeast Asia support year-round *Culicoides* activity, enabling BTV overwintering. In the past 20 years, climate change has driven BTV spread beyond 50° latitude and led to the emergence of new vector species (5). Beyan et al. and Zhugunissov et al. provided critical data on the spread of bluetongue virus (BTV) in Ethiopia and Kazakhstan, respectively. In Ethiopia, individual-level seroprevalence reached 84.5%, with age identified as a significant risk factor. In Kazakhstan, a sharp increase in BTV RNA detection occurred between 2023 and 2024, especially in goats and cattle. Infection in multiple *Culicoides* species, including *C. obsoletus*, which is recognized as a primary European vector, was confirmed by rRT-PCR. These studies reflect the dynamic expansion of BTV and the importance of regional vector competence assessments.

Despite growing interest in species distribution modeling, conceptual and methodological uncertainties are often underappreciated. While previous studies (6, 7) employed virtual species to assess the impact of sample size at comparable scales, they reported general patterns without identifying an optimal threshold. To address this gap, Mitchel et al. investigated the optimal sample size using simulated data and the Random Forest algorithm. Their findings suggest that a minimum of 750–1,000 presence/absence records is required to ensure reliable model performance, offering practical guidance for vector mapping and field sampling strategies.

Although mechanistic models have been used to examine the effects of abiotic factors on tick life cycles and population dynamics, none have explicitly modeled the mechanisms of diapause regulation (8). To fill this gap, Erguler et al. developed a structured population model that simulates tick development and diapause responses under varying photoperiod and temperature conditions. This approach improves predictions of seasonal tick activity and supports the design of targeted control measures.

Expanding the discussion to wildlife reservoirs, Han et al. identified multiple zoonotic pathogens in long-tailed ground squirrels and their ectoparasites in Xinjiang, China. DNA from *Rickettsia sibirica, R. raoultii, R. slovaca, R. felis*, and *Coxiella burnetii* was detected in both rodents and fleas/lice. This study suggests a previously underappreciated transmission cycle and emphasizes the importance of including small wild mammal reservoirs in zoonotic disease surveillance.

Patouillat et al. conducted a systematic review of zoonotic pathogen surveillance in wild Asian primates. From 152 articles, they cataloged 183 pathogens across 39 primate species. The analysis revealed strong geographic and taxonomic biases, with most studies focused on a few well-known primates and using narrow diagnostic approaches. These gaps hinder a comprehensive understanding of zoonotic spillover risks and call for a broader, One Health–oriented surveillance strategy utilizing advanced molecular techniques.

Wu et al. addressed vector control innovation through the field evaluation of a novel sugar bait device (NSBD) in four residential sites in Zhejiang, China. Combining sugar bait with adhesive capture, the NSBD trap achieved up to 86.3% reduction of adult mosquitoes and 57.8% reduction of larvae. The 10% sugar concentration yielded the most effective results. This eco-friendly, insecticide-free approach offers a promising alternative for mosquito management, particularly in urban settings with rising resistance to chemical insecticides.

Collectively, these studies highlight significant progress in vector surveillance, pathogen detection, and disease modeling, underscoring the growing importance of integrated, region-specific strategies to address the complex challenges posed by vector-borne and zoonotic diseases. The integration of high-throughput diagnostics, ecological modeling, and field epidemiology demonstrates the potential of multidisciplinary approaches to enhance early detection and response capacities.

Despite these advancements, critical gaps remain, that require sustained research investment and coordinated policy support. Adopting a One Health framework that unites human, animal, and environmental health is essential for mitigating emerging disease risks, especially in ecologically sensitive and vulnerable regions.

Moving forward, regionally adapted surveillance systems, predictive modeling, and innovative, environmentally conscious control strategies will be key components in a robust global response to evolving infectious disease threats.
